# Case report: Deciphering the clinical significance of a novel partial *BRCA1* exon 10 duplication in a patient with triple-negative breast cancer

**DOI:** 10.3389/fonc.2025.1497531

**Published:** 2025-02-06

**Authors:** Alice Faversani, Debora Manuelli, Davide Barteselli, Giulia Melloni, Carlo Santaniello, Luigi Corsaro, Davide Sacco, Davide Clerici, Laura Gargiulo, Fulvio Ferrara, Lucy Costantino

**Affiliations:** ^1^ Laboratory of Medical Genetics, Centro Diagnostico Italiano, Milan, Italy; ^2^ Department of Brain and Behavioral Science, Università Degli Studi di Pavia, Pavia, Italy; ^3^ Integrated Laboratory Medicine Services, Centro Diagnostico Italiano, Milan, Italy

**Keywords:** HBOC, breast cancer, *BRCA1*, variants of uncertain clinical significance, case report

## Abstract

Pathogenic/likely pathogenic germline variants in the *BRCA1* and *BRCA2* genes are associated with an increased risk of developing cancer, particularly breast and/or ovarian tumors. The identification and correct classification of these variants is crucial to find individuals with an increased risk of cancer and to support physicians in their clinical and therapeutic decisions. In addition, the status of *BRCA1* and *BRCA2* variants is important for appropriate management of patients’ family members. Here, we describe the case of a woman who developed triple-negative breast cancer at the age of 49 years. NGS analysis of *BRCA1* and *BRCA2* genes revealed the presence of a new partial *BRCA1* exon 10 duplication of 2.012 bp. The identified duplication comprises 395 nucleotides from the final portion of intron 9 and 1617 nucleotides from the beginning of exon 10. Using specific primers, we were able to identify the breakpoint at the DNA level and characterize the alteration as a tandem duplication leading to the formation of a premature stop codon after 10 residues. RNA analysis allowed to confirm the production of an altered mRNA showing the duplicated sequence. In this way, we were able to assign a clinical significance to the new alteration and classify it as a pathogenic variant. Although new ClinGen ENIGMA guidelines have been produced to provide tools for the accurate interpretation of variants in the *BRCA1* and *BRCA2* genes, defining the clinical significance of copy number variants, particularly duplications, remains a challenging goal that requires complex approaches to accurately determine the role of such variants. Other investigations, such as the detection of breakpoints by RNA analysis, are often essential to classify the identified alteration. Our study suggests that RNA transcript analysis is an ideal methodology to support the accurate classification of variants and clarify their effects.

## Introduction

Hereditary Breast and Ovarian Cancer Syndrome (HBOC) is characterized by an increased incidence of breast cancer in both men and women, ovarian cancer, and other tumors such as pancreatic and prostate cancer (1). HBOC is inherited in an autosomal-dominant manner, resulting in a lifetime risk of 50-80% for breast cancer and 30-50% for ovarian cancer (2). Therefore, early identification of carriers is crucial for patient stratification and helps physicians in determining the most suitable surveillance, treatment strategies, and follow-up program.

*BRCA1* and *BRCA2* are tumor suppressor genes that ensure genomic stability as they are involved in the repair of DNA double-strand breaks by homologous recombination (3).

Next Generation Sequencing (NGS) analysis of *BRCA1* and *BRCA2* is used to identify individuals with germline variants in these genes and with an increased risk of *BRCA*-related tumors. Classification and characterization of the variants identified through NGS technology are critical in determining their pathogenicity and clinical significance. However, the detection of variants of uncertain significance (VUS) limits the clinical utility of such testing (4). The surge in clinical germline testing has resulted in a significant increase in the detection of VUS, encompassing both copy number variations and point mutations.

Copy number variants (CNVs) account for between 5-10% of the human genome (5, 6). They are responsible for most of the variation in the human genome as they can alter gene structure, function and expression (6). Germline CNVs located within the *BRCA1* and *BRCA2* gene loci are associated with the development of breast cancer and account for 6-10% of known pathogenic variants in these genes (7, 8). Recently, new ClinGen ENIGMA (Evidence-based Network for the Interpretation of Germline Mutant Alleles) *BRCA1* and *BRCA2* variants interpretation guidelines have been published (9). Nevertheless, CNVs classification, especially duplications, remains highly controversial and difficult even today due to the lack of adequate tools that would facilitate a clear classification of these variants.

Variants of uncertain significance and their effects remain one of the unsolved issues, as their impact on increasing risk is unpredictable. Functional studies make it possible to characterize these variants so that their true pathogenicity can be defined.

In this study, we report the case of a female patient who developed triple-negative breast cancer at the age of 49. *BRCA1* and *BRCA2* analysis revealed the presence of a partial duplication of the *BRCA1* exon 10. Only the identification of the breakpoint at the DNA level and the RNA analysis allowed us to determine the clinical significance of the reported CNV.

## Case description

This patient was a 50-year-old woman with a personal history of breast cancer who underwent genetic counselling at our medical genetics clinic at Centro Diagnostico Italiano in Milan, Italy.

At the age of 49, she complained about mastodynia at the left gland which was the reason for performing a bilateral mammography and ultrasound. Two lumps, measuring 20 and 23 mm, were retrieved at the left breast.

A core-needle biopsy was performed, and histologic examination revealed: for the 23 mm lump, a No Special Type Invasive Carcinoma, Grade 3, Estrogen Receptor 0%, Progesterone Receptor 0%, Ki67 39% HER2 score 0; for the 20 mm nodule, a No Special Type Invasive Carcinoma, Grade 3, Estrogen Receptor 5%, Progesterone Receptor 0%, Ki67 65% HER2 score 0.

After performing instrumental evaluation (PET, Positron Emission Tomography), cancer staging was cT3N0M0. Neoadjuvant chemotherapy with four cycles of Carboplatine and Taxol and four cycles with Adriamycin Cyclophosphamide and immunotherapy with Pembrolizumab was planned.

However, neoadjuvant chemotherapy with Adriamycin Cyclophosphamide and Pembrolizumab was discontinued after three cycles due to immunotherapy- related toxicity, followed by left mastectomy.

Histology revealed No Special Type Invasive Carcinoma, Grade 3, Estrogen Receptor negative, Progesterone Receptor negative, Ki67 35% HER2 score 0; ypT2 (3.5 cm) N0 (0/9).

Adjuvant chemotherapy with Capecitabine for six months and local radiotherapy followed the surgery.

One year after mastectomy, multiple metastases were identified in the lungs and mediastinal lymph nodes, thus Carboplatin and Gemcitabine adjuvant chemotherapy was started. After six months, treatment was shifted to Sacituzumab/Govitecan due to an allergic reaction to Carboplatin.

**Figure 1:**
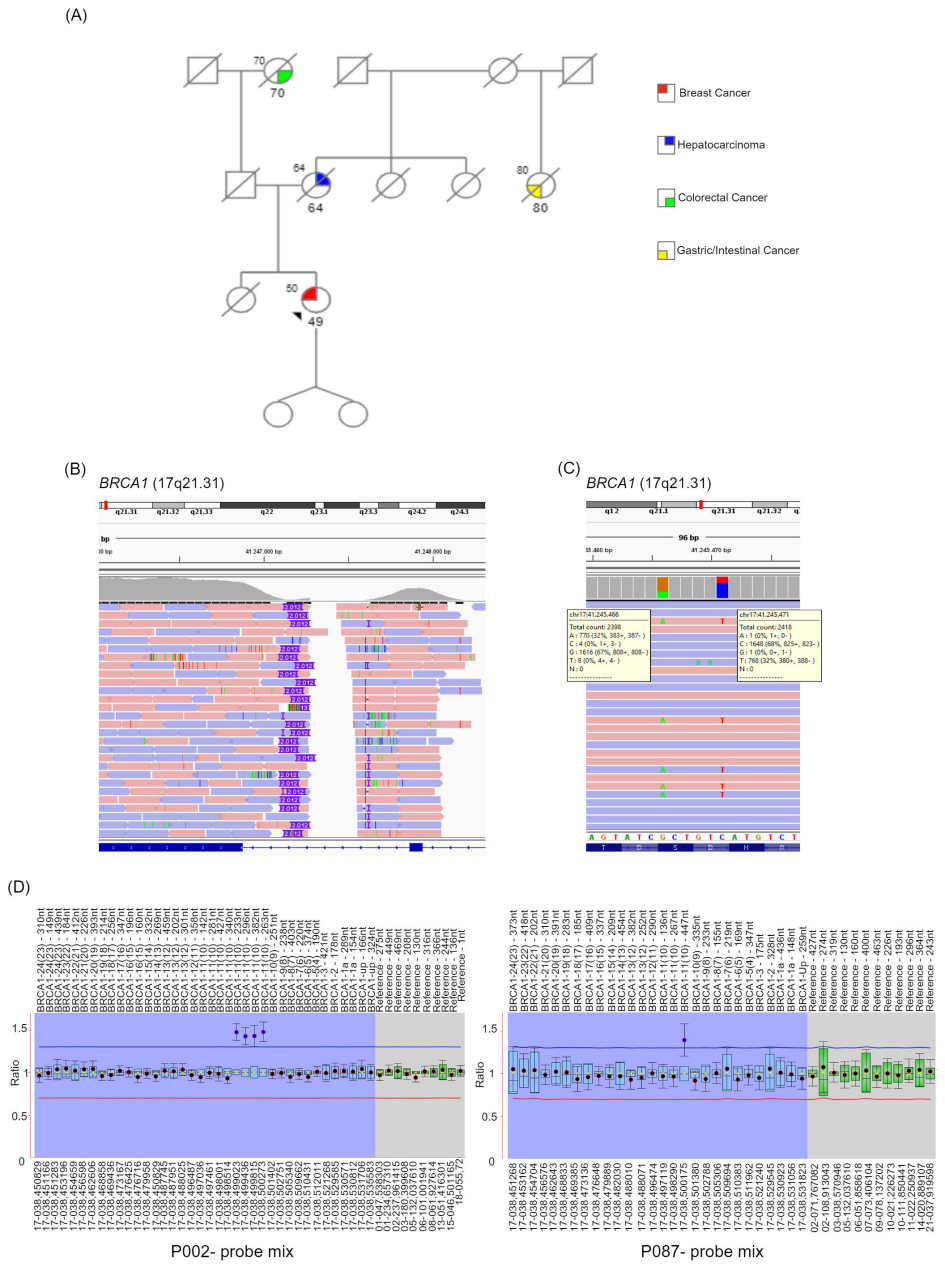
Patient’s family pedigree, NGS and MLPA analysis. **(A)** The pedigree of the patient’s family is shown. Females and males are indicated as circles and squares, respectively. The arrow indicates the proband. The type of cancer affecting family members is displayed in the legend. The age at the tumor diagnosis is reported under the corresponding symbols. Diagonal lashes indicate deceased individuals, and the age at the time of death is indicated on the top left of the corresponding symbol. For the proband only, the age at the testing time is reported at the top left of the corresponding symbol. Patient’s NGS analysis results are shown by IGV: **(B)** highlights the presumed insertion of 2012 nucleotides; **(C)** shows the two benign variants localized at the exon 10 of *BRCA1* and identified with a variant fraction of about 30%; **(D)** MLPA analysis has been performed to investigate the presence of a *BRCA1* exon 10 partial duplication. The panel shows MLPA results using the P002 and P087 probe mix. *BRCA1* exons are numbered according to the legacy numbering. The current exon numbering is given in brackets.

As shown in **Figure 1A**, she had a sister who died, at 18 years of age, for reasons unrelated to cancer. Her mother had hepatocellular carcinoma and died of it at 64 years old. A maternal aunt was reported to have been diagnosed with gastrointestinal cancer at the age of 80 (**Figure 1A**). The mother had two sisters, who died at an advanced age for non-cancerous causes (**Figure 1A**).

On the paternal side, the grandmother had colorectal cancer at the age of 70 years, while the father died of an infection at the age of 47 (**Figure 1A**). Two paternal uncles (only maternal side) died for reasons unrelated to cancer in their 50s-60s.

She has two 29-year-old daughters, who are monozygotic twins and cancer-free.

The clinical data made the patient eligible for genetic testing according to the criteria Associazione Italiana di Oncologia Medica (AIOM) 2021 (10). After this evaluation, the patient signed the informed consent, and blood samples were collected to analyze *BRCA1* (NM_007294.4) and *BRCA2* (NM_000059.4) genes.

Next Generation Sequencing analysis of these genes using an amplicon-based technique (CE-IVD NGS *BRCA* Devyser kit, Devyser, Sweden) did not reveal any single nucleotide pathogenic/likely pathogenic variants or variants of uncertain significance, but showed an altered CNVs analysis, suggesting a duplication at *BRCA1* exon 10 (*BRCA1* legacy exon 11), as the data in this region were borderline of the normal range (**Supplementary Figure S1**). To clarify the CNVs profile, we performed a second NGS analysis with a different technology, a capture-based kit (CE-IVD SOPHiA DDM™ Dx Hereditary Cancer Solution, Boston, USA). This technique did not reveal an altered CNVs status. On the other hand, the Integrative Genomics Viewer (IGV) software, using the obtained NGS capture-based data, showed an insertion of 2012 nucleotides at the *BRCA1* exon 10 (**Figure 1B**). Additionally, both procedures identified two benign variants, NM_007294.4:c.2077G>A NP_009225.1:p.(Asp693Asn) and NM_007294.4:c.2082C>T NP_009225.1:p.(Ser694=), with a variant fraction of about 30% (**Figure 1C**) localized in *BRCA1* exon 10. All these data suggested the presence of a potential genetic rearrangement within exon 10 of the *BRCA1* gene.

To confirm the diagnostic suspicion, Multiplex Ligation-dependent Probe Amplification (MLPA) was first performed using DNA isolated from a second blood sample and the P002 probe mix (MRC-Holland, Amsterdam, Netherlands). The MLPA analysis revealed a duplication in *BRCA1* exon 10 (**Figure 1D**, **Supplementary Figures S2A**, **S2B**). Notably, four out of eight probes in this region showed values outside the normal range, as indicated in **Figure 1D**. The P087 probe mix was then used as a confirmatory probe for the MLPA results (**Figure 1D**).

Based on the MLPA analysis, we concluded that the patient harbored a heterozygous partial duplication of the *BRCA1* exon 10, which, based on MLPA probes positions, involved the first part of the exon 10. Specifically, the 3’ ligation site position of the last altered probe was located 1337 nucleotides from the start of exon 10, while the 3’ ligation site of the next probe with a normal copy number was 1847 nucleotides from the start of the exon (**Supplementary Figure S2**). However, we were unable to identify the breakpoints and determine the clear clinical relevance of the identified alteration.

**Figure 2:**
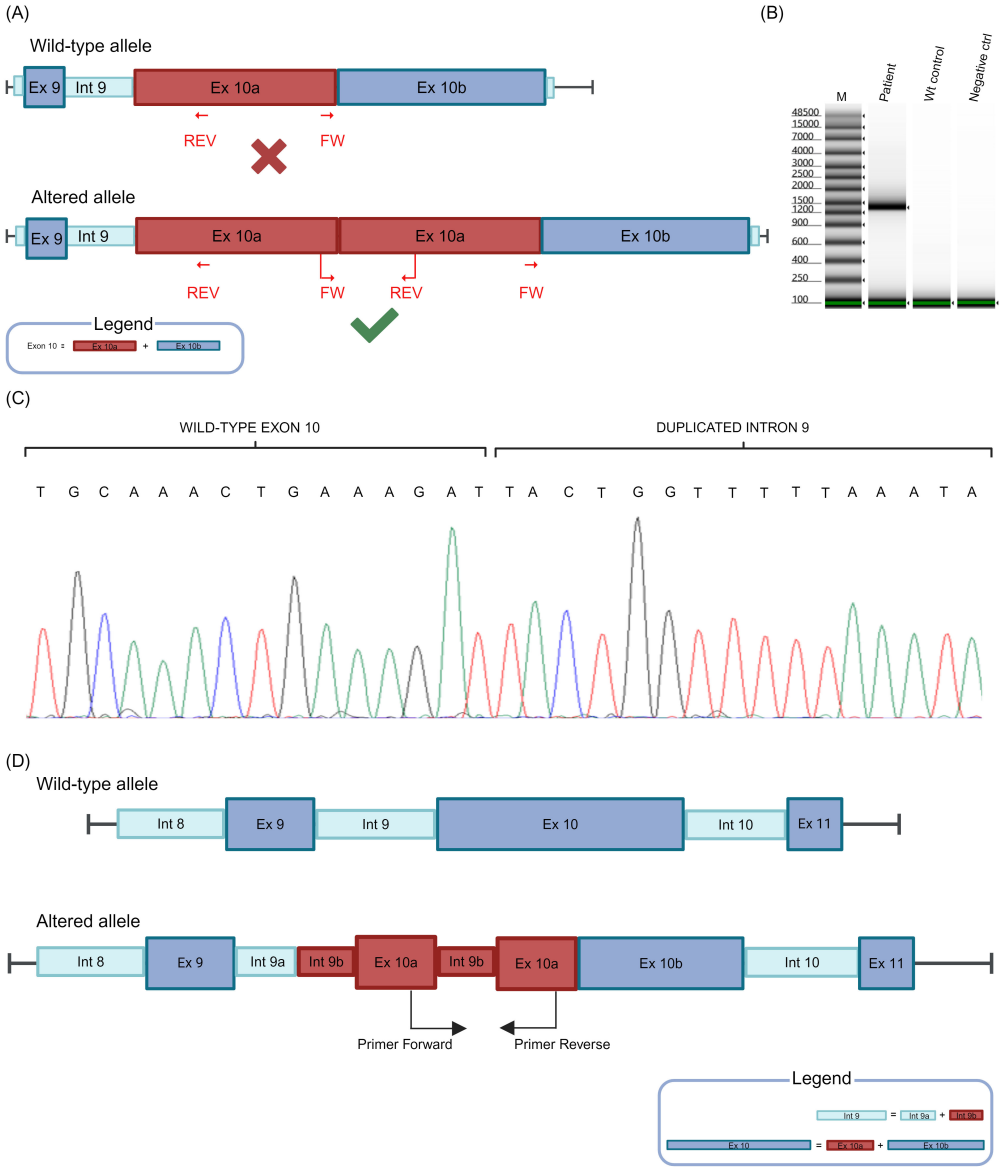
PCR analysis and Sanger sequencing, using specific primers able to amplify only the altered allele, allowed to identify the exact breakpoint and the duplicated sequence. **(A)** A schematic representation of the designed primers is reported for wild-type and the altered alleles we had supposed. The possibility of obtaining or not a PCR product is represented with the symbols V and X, respectively. To better understand the graphic representation of the possible partial duplication of *BRCA1* exon 10, this exon has been divided into two segments (Ex 10a and EX 10b) (Intron, Int and Exon, Ex). **(B)** Electrophoresis of the amplicons obtained using the designed primers. Only the altered allele can be amplified. **(C)** Sequencing chromatogram shows the breakpoint and the intronic DNA sequence introduced immediately after. **(D)** Schematic representation of the patient’s *BRCA1* exon 10 partial duplication, compared to the wild-type *BRCA1* gene (Intron, Int and Exon, Ex). To facilitate the understanding of the identified duplication, Intron 9 and Exon 10 have been divided into two segments: Int 9a and Int 9b and Ex 10a and Ex 10b, respectively.

To determine the exact breaksite and sequence of the duplication, presuming a tandem duplication, we designed forward and reverse primers able to amplify only the altered allele (**Figure 2A**, **Supplementary Table S1**). The designed forward primer was positioned at the last MLPA probe detected outside the range within exon 10, while the reverse primer was located at the start of the exon 10 (**Figure 2A**). This strategy allowed the amplification of the only allele harboring the duplication and not of the wild-type allele in case of tandem duplication (**Figure 2A**).

When we performed PCR with the created primers pair, we compared the results obtained with the DNA derived from the patient and a *BRCA1* wild-type subject. We analyzed these samples using the Genomic DNA ScreenTape Analysis Kit (Agilent Technologies, Santa Clara, CA, USA). As shown in **Figure 2B**, only the patient’s DNA revealed a PCR product, while no amplification was observed for the *BRCA1* wild-type control (**Figure 2B**). We then performed Sanger sequencing on this product, which allowed the identification of the breaksite and the DNA sequence introduced immediately after (**Figure 2C**). Careful analysis of the electropherogram made possible to define that the duplicated sequence contained part of the intron (395 bp) between exon 9 and exon 10 and that the size of the duplicated sequence, which amounted to 2.012 base pairs, was composed of 395 bp from the intron and 1617 bp from the exon 10 (**Figure 2D**).

The characterization of the identified variant made it be possible to describe the alteration as: NC_000017.11:g.43093244_43095255dup NG_005905.2(NM_007294.4):c.671-395_2287dup. This sequence suggests a stop codon (TAA) insertion after 10 residues at the protein level: NP_009225.1:p.(Ser763Leufs*10).

Despite the variant leads to a premature stop codon in a region where Nonsense-Mediated Decay (NMD) is predicted, we inferred the wild-type protein (1863 amino acids) and the one that could be generated following the identified variant (**Supplementary Figure S3**), using the AlphaFold platform (https://alphafold.ebi.ac.uk). The altered protein would consist of 771 amino acids and lack potentially clinically relevant regions, such as the Coiled-Coil and BRCT (BRCA1 C-terminal region) domains. A spatial comparison of the two proteins yielded a Weighted RMSD of 0.332 Å.

To understand the functional impact of this alteration, we performed *in silico* predictions of pathogenicity and of splicing process. To assess the pathogenicity of the identified duplication, we utilized Python packages that calculated pathogenicity probability. The input for this analysis was provided in bed file format with the variation type specified as duplication. The packages used for this analysis were ClassifyCNV (11), ISV CNV (12), and StrVctVre (13). For duplication annotation, we used the AnnotSV platform (https://lbgi.fr/AnnotSV/). The predictions are summarized in the **Supplementary Table S2**. Further analysis using the AnnotSV portal indicated there are 1996 overlapping pathogenic sequences. The *in silico* prediction of potential alterations in the splicing process performed using the Berkeley Drosophila Genome Project (BDGP) tool highlighted the duplication of the acceptor and donor sites in the region affected by the variant and the creation of a new acceptor site downstream of the breakpoint. Moreover, the SpliceAI (https://spliceailookup.broadinstitute.org/) tool predicted an acceptor gain with a high precision score (0.98) within the duplicated sequence (14).

**Figure 3:**
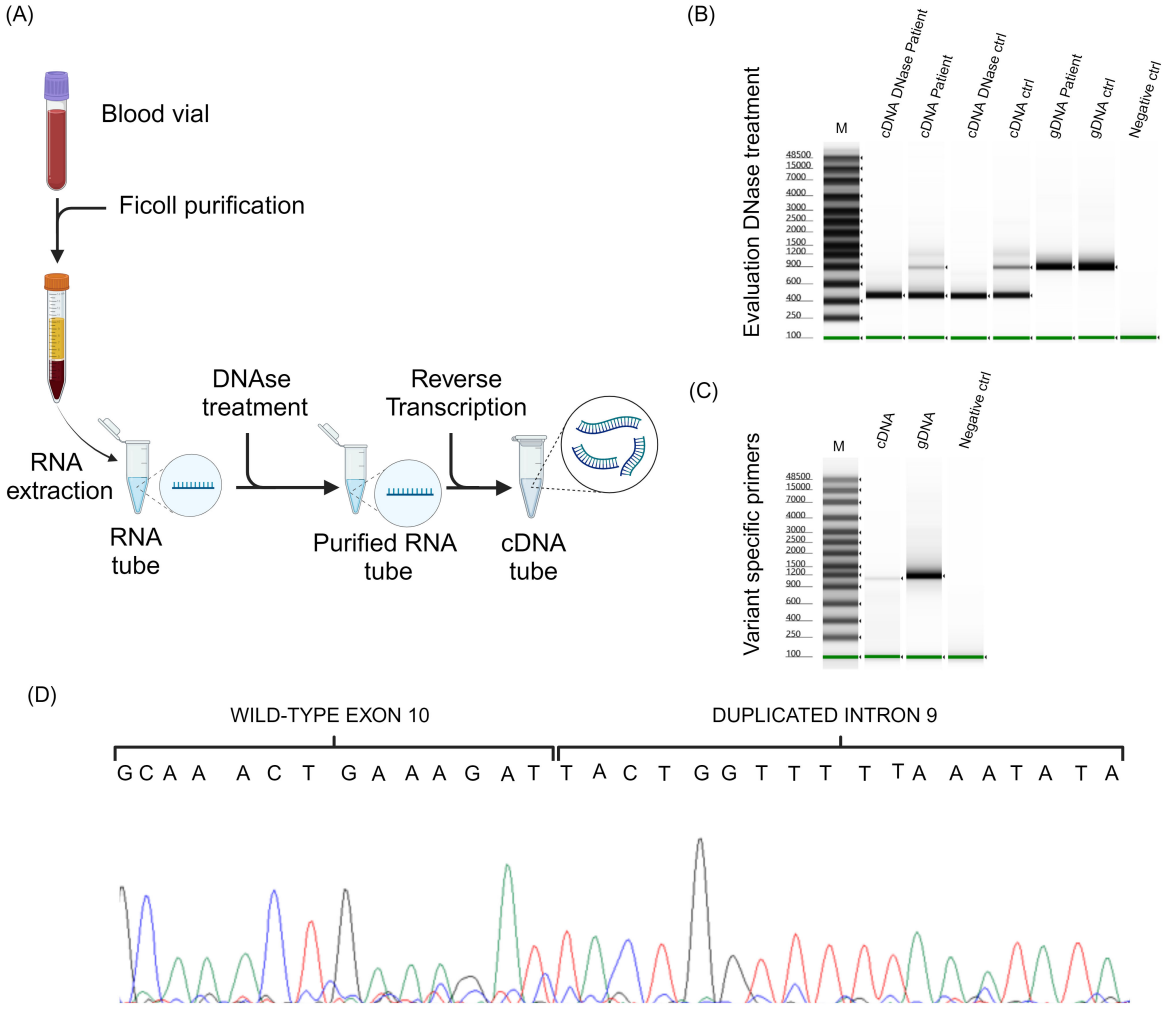
RNA analysis. **(A)** Schematic representation of patient’s peripheral blood mononuclear cells isolation, RNA purification and reverse transcription. **(B)** The result of the PCR analysis performed to verify the DNase treatment. **(C)** Electrophoresis of the amplicons obtained using the designed primers able to amplify only the altered allele. **(D)** Shows the sequencing chromatogram obtained by cDNA analysis.

To determine whether this duplication also had an impact at the RNA level, the patient’s peripheral blood mononuclear cells were isolated from blood samples by stratification with Ficoll-Paque PLUS (Eppendorf, Hamburg, DE), and RNA was purified using QIAamp RNA Blood Mini kit (Qiagen, Hilden, DE), as illustrated in **Figure 3A**. To avoid any DNA contamination, RNA was treated with DNase I (**Figure 3A**, Thermo Fisher Scientific, Whaltam, USA) and reverse transcription was performed using High-Capacity cDNA (complementary DNA) Reverse Transcription Kits (**Figure 3A**, Thermo Fisher Scientific, Whaltam, USA).

To confirm the absence of genomic DNA contamination in the samples after digestion with DNase I, we performed a PCR using primers that produce an amplified product containing *BRCA1* intron 10 from genomic DNA (**Supplementary Table S1**). To test this hypothesis, we used both the cDNA from our patient and the cDNA from a control subject. The cDNA derived from both patient and control RNA treated with DNase I were compared to the cDNA derived from untreated RNA. To strengthen the data and ensure that the PCR product was identical to that produced from genomic DNA, we also included the two genomic DNA samples that underwent the same PCR.

According to primers positions, the expected PCR product sizes were 458 bp for samples without intron 10 and 860 bp in those with it. As shown in **Figure 3B**, the PCR products derived from the DNA samples exhibited a size consistent with the presence of intron 10; the amplicons derived from cDNA without DNase I treatment showed 2 different PCR fragments, one approximately 458 bp and one 860 bp, compatible with the presence of residual genomic DNA in the sample; whereas the presence of a single band of 458 bp in the cDNA samples, both from the patient and the control treated with DNase I, was attributable only to the presence of exonic sequences (**Figure 3B**). To better appreciate the PCR product size, we analyzed these samples using the Genomic DNA ScreenTape Analysis Kit (Agilent Technologies, Santa Clara, CA, USA).

The cDNA was then processed by PCR using the same primers as for DNA analysis (**Supplementary Table S1**). PCR was performed with the patient’s DNA and cDNA (**Figure 3C**). To better appreciate PCR products size, we analyzed these samples using the Genomic DNA ScreenTape Analysis Kit. Sanger sequencing (**Figure 3D**) allowed to determine the effective sequence of RNA after transcription. cDNA sequence showed all the duplicate sequence identified at the DNA level, also the duplicate intron portion.

These findings indicated an out-of-frame tandem duplication, resulting in a frameshift and a premature stop codon, with changes in protein production, p.Ser763Leufs*10. **Supplementary Figure S4** shows the DNA and cDNA chromatograms with the breakpoint and the formation of a stop codon after 10 residues from the insertion of the duplicated nucleotides. Thanks to this methodological approach, we were able to classify the newly identified CNV as pathogenic (Class 5) according to the ClinGen ENIGMA *BRCA1* and *BRCA2* Expert Panel Specifications to the ACMP/AMP Variant Interpretation Guidelines for *BRCA1* (9).

To explore the mechanisms underlying the identified duplication, we investigated the presence of Short Interspersed Nuclear Elements (SINEs), and Long Interspersed Nuclear Elements (LINEs) located in the region involved in the patient’s CNV. Analyzing the *BRCA1* DNA sequence from the beginning of exon 8 (g.43097289) to the end of intron 11 (g.43082576), using RepeatMasker v4.0.9 (https://www.repeatmasker.org/), we identified 17 SINEs (*Alu* elements or Mammalian-wide Interspersed Repeats, MIRs), 10 LINEs (L1 and L2) and 1 Long Terminal Repeat (LTR), in both wild-type and altered *BRCA1* sequences (**Table 1**). An *AluJB* element has been highlighted at 44 nucleotides upstream of the duplicated region. No SINEs, LINEs or LTR have been found at the end or into the duplicated sequence. No new SINEs, LINEs or LTR have been formed by the *BRCA1* sequence alteration.

**Table 1:** SINEs and LINEs analysis.

Begin Wild Type Sequence (Altered Sequence) §	End	Repeat	Repeat Class/Family	Score
135 (135)	313 (313)	L1PREC2	LINE/L1	780
330 (330)	399 (399)	MIR3	SINE/MIR	343
700 (700)	869 (869)	AluSx	SINE/Alu	1165
884 (884)	1194 (1194)	AluSx	SINE/Alu	2271
1679 (1679)	1990 (1990)	AluJb	SINE/Alu	2026
6356 (8368)	6388 (8400)	(GT)n	Simple_repeat	16
6462 (8474)	6558 (8570)	L2d2	LINE/L2	222
6641 (8653)	6917 (8929)	AluSx	SINE/Alu	2022
7015 (9027)	7086 (9098)	MIRb	SINE/MIR	263
7260 (9272)	7562 (9574)	AluJb	SINE/Alu	1799
7618 (9630)	7744 (9756)	AluSx	SINE/Alu	898
7957 (9969)	8259 (10271)	AluSx	SINE/Alu	1994
8333 (10345)	8459 (10471)	L2d2	LINE/L2	206
8470 (10482)	8558 (10570)	MIRb	SINE/MIR	192
8750 (10762)	8846 (10858)	L1MB3	LINE/L1	604
8856 (10868)	8906 (10918)	MER66B	LTR/ERV1	320
8925 (10937)	9121 (11133)	L1MB3	LINE/L1	1029
9240 (11252)	9549 (11561)	AluJb	SINE/Alu	1153
9599 (11611)	9909 (11921)	AluSx	SINE/Alu	2293
9913 (11925)	10267 (12279)	L1ME4a	LINE/L1	651
10956 (12968)	11041 (13053)	L2c	LINE/L2	366
11142 (13154)	11180 (13192)	(TG)n	Simple_repeat	46
11184 (13196)	11295 (13307)	L1ME2z	LINE/L1	457
11615 (13627)	11751 (13763)	L2c	LINE/L2	205
11907 (13919)	12032 (14044)	AluSx	SINE/Alu	877
12826 (14838)	12960 (14972)	AluSx	SINE/Alu	2258
12961 (14973)	13258 (15270)	AluY	SINE/Alu	2438
13259 (15271)	13432 (15444)	AluSx	SINE/Alu	2258
13708 (15720)	13771 (15783)	L2b	LINE/L2	247
13835 (15847)	14131 (16143)	AluSx	SINE/Alu	1819

The table shows the SINEs, LINEs and LTR sequences identified within the *BRCA1* DNA region, spanning from the start of exon 8 (g.43097289) to the end of intron 11 (g.43082576).

§: the position of the identified element into the wild type and altered DNA sequence is reported. The position into the altered DNA sequence is given in brackets. Position 1 corresponds to the nucleotide located on g.43097289.

The last column SCORE reports the outcome of the Smith-Waterman algorithm is used for local sequence alignment, which identifies regions of similarity between two sequences. The score resulting from a Smith-Waterman alignment provides a measure of similarity between segments of the sequences. Higher Scores: Indicate a greater degree of similarity between the aligned segments of the sequences. This suggests that the two sequences share more matching or similar sub-sequences. Lower Scores: Indicate less similarity. If the score is very low or zero, it suggests there are few to no significant matching sub-sequences between the two sequences.

## Discussion

In this study, we discuss the case of a patient affected by triple-negative breast cancer who was found to have an uncharacterized partial duplication in *BRCA1* exon 10 corresponding to *BRCA1* legacy exon 11.

When we performed NGS analysis for *BRCA1* and *BRCA2* genes, the CNV analysis suggested a possible rearrangement, although the signals were borderline. Additionally, two benign *BRCA1* variants, localized in *BRCA1* exon 10 were identified, each with a low variant allele frequency. These data led us to suspect a possible genetic rearrangement within *BRCA1* exon 10. This doubt was clarified by MLPA analysis, which allowed us to understand that the first 1379 nucleotides of *BRCA1* exon 10 were duplicated. However, we had no data to identify the breakpoints and the entire duplicated DNA sequence. It was also unclear whether the duplicated region was in tandem and if it disrupted the normal reading frame, introducing a premature STOP codon. Exon 10 is the largest exon of *BRCA1* but does not encode any clinically important residues or functional protein domains.

Therefore, with the data collected thus far, we could not determine the clinical significance of the identified CNV. Further experiments were needed to ascertain the size of the duplicated sequence and its potential impact on BRCA1 protein function. Due to the limitations of classical DNA analysis in precisely determining the clinical impact of the identified variant, we chose to apply a transcript analysis model. This approach allowed us to further investigate the genetic rearrangement in exon 10 and to determine the reading frame and tandem status of the *BRCA1* gene.

Using a primers combination specific for the reported variant, we identified it as a tandem duplication involving 2012 nucleotides: 395 bp derived from the neighboring *BRCA1* intron 9 and 1617 bp from the first part of exon 10.

We performed RNA analysis to determine if the duplicated sequence was retained in the mature mRNA, and the entire duplicated region was detected at the RNA level.

Through these experiments, we demonstrated that the identified CNV led to the formation of a premature stop codon 10 residues away from the insertion site. As previously reported in the literature (7), the formation of a premature stop codon triggers degradation of the RNA molecule by the NMD process. This process prevents adequate protein production, which likely determines the pathogenicity of this variant. For these reasons, we interpreted the identified CNV as a pathogenic variant (Class 5).

Indeed, according to ClinGen-ENIGMA *BRCA1* and *BRCA2* Expert Panel Specifications to the ACMP/AMP Variant Interpretation Guidelines for *BRCA1* (9), we assigned the following interpretation criteria to the reported variant: PVS1_Very Strong, null variant predicted to result in NonSense Mediated Decay (NMD); PM5(PTC)_Strong, formation of a Protein Termination Codon (PTC) at the exon 10. We are able to assign the PVS1_Very Strong and not the PVS1 (RNA), at the moment. Additional functional investigations are needed for the use of PVS1 (RNA) criteria. We didn’t assign the PM2_Supporting criterion, as its use is not recommended for insertion, deletion or insertion-deletion variants due to their estimated low recall (15).

The classification of genetic variants, particularly those involving duplications, is crucial for understanding their potential pathogenicity. In our analysis, we employed various bioinformatics tools, such as Classify CNV, ISV CNV, and StrVctVre, which provided a systematic approach to assess the likelihood that the identified duplication contributes to disease. This computational framework is useful, as it integrates multiple data sources and algorithms to offer a comprehensive view of the variant’s implications.

The pathogenicity assessment revealed that the protein product derived from the rearrangement may be non-functional, potentially impacting cellular processes. This conclusion is supported by the findings from the AnnotSV platform, which we used for annotation. The classification of the variant, although it does not provide an unequivocal opinion on the classification and impact of the damage, underscores the complexity of the genetic alterations and their potential roles in disease mechanisms.

Furthermore, *in silico* predictions conducted with the BDGP and SpliceAI tools indicated critical alterations in the splicing process (14). Notably, the duplication impacted both the acceptor and donor sites within the affected region and resulted in the formation of a new acceptor site downstream of the breakpoint. These changes may disrupt normal splicing patterns, leading to aberrant protein products that could contribute to pathogenicity.

These findings align with existing literature on the implications of similar genetic duplications. For instance, studies have shown that alterations in splicing can lead to various genetic disorders (16). Such evidence reinforces our conclusion that the protein product arising from the rearrangement is likely to be non-functional, supporting the pathogenicity of the identified duplication.

This scenario becomes even more significant when considering that *BRCA1* gene has numerous mRNA splicing isoforms (17). In particular, exon 10 (Legacy exon 11) exhibits different alternative splicing events, known in literature as follows: Δ11, with the skipping of the exon 10; Δ11q with partial exon 10 skipping for the use of an alternative donor site within the exon 10; Δ9, 10, 11q with exon 8, 9 and partial exon 10 skipping; IRIS, with the skipping of the exon 12-24, but retention of short region of the intron 10 (Legacy intron 11) (18). These isoforms are involved in physiological and pathological processes such as tumor development (18). However, the balance among these isoforms appears crucial for cell fate. Alteration of the natural ratio between *BRCA1* isoforms could be involved in cancer predisposition. The formation of an alternative acceptor site, due to the presence of the newly identified variant, could lead to alteration of the splicing process and cause a disruption in the balance between the different *BRCA1* isoforms, altering their physiological equilibrium.

The assay, that we used, highlighted the formation of a transcript in which was presence the duplicated region leading to the formation of a premature stop codon. However, using this assay we were not able to perform allele-specific quantification and to analyze the different *BRCA1* isoforms. For this reason, we cannot know if the natural balance between *BRCA1* exon 10 isoforms is disrupted or if other RNA products could be formed by the presence of the identified variant. Further investigations, including *in vitro* functional experiments and RNA sequencing, are needed to determine if this variant induces splicing alterations.

To find a possible cause for the formation of the identified CNV, we investigated the presence of Short Interspersed Elements (SINEs), such as *Alu*, and Long Interspersed Elements (LINEs) from the beginning of *BRCA1* exon 8 to the end of *BRCA1* intron 11. These highly repetitive DNA sequences contribute to genomic instability and are involved in mechanisms that cause CNVs (8). The *BRCA1* gene shows a great number of *Alu* elements that may be responsible for *Alu*-*Alu* mediated CNVs (19). Our analysis highlighted only one *AluJB* element at 44 nucleotides upstream at the beginning of the duplicated DNA region. No other repetitive sequences were identified at the end of the duplicated region, suggesting that the identified CNV is unlikely to be *Alu*-*Alu* mediated.

Pathogenic germline variants in the *BRCA1* and *BRCA2* genes predispose individuals to HBOC syndrome. Most pathogenic variants identified are single nucleotide alterations, including base substitutions, small deletions/duplications and insertions. These variants affect protein production leading to protein truncation, NMD and amino acids substitutions that impact the protein function. Large rearrangements, such as large deletions or duplications, are less common (20, 21) and account for approximately 10-15% of all variants identified in *BRCA1* (19).

The clinical interpretation of large rearrangements is often challenging, especially for large duplications, which, without additional pathogenetic evidence, are typically classified as VUS due to the lack of information about the duplication length and status (22). Large exonic deletions are usually pathogenic as they disrupt the reading frame leading to the formation of a premature STOP codon or result in the loss of important functional regions. Instead, it is much more difficult to decipher the clinical significance of large duplications as they can lead to disrupt the reading frame or the natural splicing process, but it is mandatory to understand the breakpoints and the orientation of the duplicated region. According to ClinGen-ENIGMA *BRCA1* and *BRCA2* Expert Panel Specifications, to interpret the clinical significance of large duplications involving one or more exons of *BRCA1*, it is necessary to determine whether the variant disrupts the reading frame or if the frame is maintained. Additionally, the tandem status (proven or presumed) of the duplication is crucial. The entire process becomes more complicated when, as in our case, only part of an exon is involved in the duplication, rather than the entire exon. In this case, it is essential to determine if it creates a frameshift or not and to establish the breakpoints.

NGS and MLPA analyses can identify the presence of large duplications; however, they do not provide data on the tandem status of the extra copies (23). To understand whether a duplication is in tandem or translocated to another region of the genome, it is essential to determine its effects on the reading frame and the formation of a premature STOP codon. Using an RNA analysis model allows for a detailed understanding of the RNA transcriptome, which can significantly enhance the characterization and classification of genetic variants. This approach enables the identification of aberrant splicing events, the detection of alternative transcripts, and the evaluation of the presence and effects of novel mutations at the RNA level (24). By analyzing RNA, we can directly observe the impact of genetic variations on transcript production and stability, thereby providing a more comprehensive assessment of variant pathogenicity. This method is particularly valuable in cases where DNA analysis alone cannot elucidate a variant’s clinical significance.

Our study has characterized a novel *BRCA1* exon 10 partial duplication using RNA analysis. Only through this method we could attribute a clinical significance to the detected CNV, reclassifying it from a VUS to a pathogenic variant.

Detecting a VUS leaves patients and their physicians with an inconclusive genetic result without a proper clinical diagnosis. Reclassifying a VUS strongly impacts patient clinical management, allowing access to prevention programs and helping to predict responses to targeted therapies. In particular, the reclassification of this novel variant allows the patient to be treated with PARP-inhibitors, since only pathogenic/likely pathogenic variants grant access to this therapy, which, thanks to the Olympia study (25), is now offered to patients with HER2 negative breast cancers, especially in the metastatic stage.

In addition, detecting pathogenic variants is crucial for patients’ families, as it enables the early identification of at-risk individuals who may benefit from clinical surveillance. In this family, the result allows the patient’s daughters to be tested for the familial variant. They have not been tested yet due to logistical issues; however, if they are not carriers of the familial variant, they can be considered to have the same oncologic risk as general population. Conversely, if they have inherited their mother’s variant, they can access intensive surveillance programs (every six months) or consider risk reduction mastectomy/adnexectomy.

This reclassification of this variant is also valuable for future patients who may carry an identical, or similar variant. A patient with an identical variant could have direct access to PARP-inhibitor therapy or bilateral mastectomy/adnexectomy if needed.

The frequency of VUS in the *BRCA1* and *BRCA2* genes is about 5-15%, including less studied populations (26–28). The introduction of the ClinGen ENIGMA guidelines specific for *BRCA1* and *BRCA2* (9) has enabled the interpretation of a large number of variants previously classified as VUS (29). However, a significant proportion of VUS still remains without biological significance due to limited experimental and clinical data. CNVs, especially large duplications, are variants that require robust experimental and functional studies for accurate interpretation.

Further studies are needed to clarify the role of the identified variant in HBOC, such as segregation analysis of this novel pathogenic variant within the patient’s family. Additionally, *in vitro* experiments would be required to better assess its biological effect, including potential alterations in the physiological splicing process and its impact on the pathways in which BRCA1 is involved.

Clarifying the splicing process in this *BRCA1* region may be helpful in classifying variants within the same region. In fact, the splicing process of *BRCA1* exon 10 is known to be complex, producing different natural isoforms (17). In this family we cannot rely on segregation analysis and the absence of other breast/ovarian cancers may suggest a paternally inherited variant, a maternally variant with reduced penetrance, or, although rare (30), a *de novo* occurrence of this new variant. In this context, studying the different *BRCA1* exon 10 isoforms in our case may add knowledge not only to our variant but also to the debated clinical significance of variants affecting the exon 10 boundary.

In conclusion, our work highlighted the importance of RNA analysis in reducing the impact of VUS detection in genetic analyses and the utility of integrated bioinformatics approaches in elucidating the pathogenicity of genetic variations. This comprehensive assessment enhances our understanding of the molecular mechanisms underlying genetic disorders and can help improving patient’s clinical management.

## Data Availability

The original contributions presented in the study are included in the article/**Supplementary Material**. Further inquiries can be directed to the corresponding author.
