# Supplementary material for: Case report: Deciphering the clinical significance of a novel partial BRCA1 exon 10 duplication in a patient with triple-negative breast cancer
Source: Front Oncol. 2025 Feb 6;15:1497531. doi: 10.3389/fonc.2025.1497531 (PMC11839443; doi:10.3389/fonc.2025.1497531)
Supplement: Supplementary file 1 [file DataSheet1.docx]

**Case report: Deciphering the clinical significance of a novel partial *BRCA1* exon 10 duplication in a patient with triple-negative breast cancer**

Alice Faversani^1#^, Debora Manuelli^1#,^ Davide Barteselli^1#^, Giulia Melloni^1^, Carlo Santaniello^1^, Luigi Corsaro^1,2^, Davide Sacco^1,2^, Davide Clerici^1^, Laura Gargiulo^1^, Fulvio Ferrara^3^, Lucy Costantino^1*^

^#^These authors contributed equally to this work

* Correspondence:
 Lucy Costantino
 [lucy.costantino@cdi.it](mailto:lucy.costantino@cdi.it)


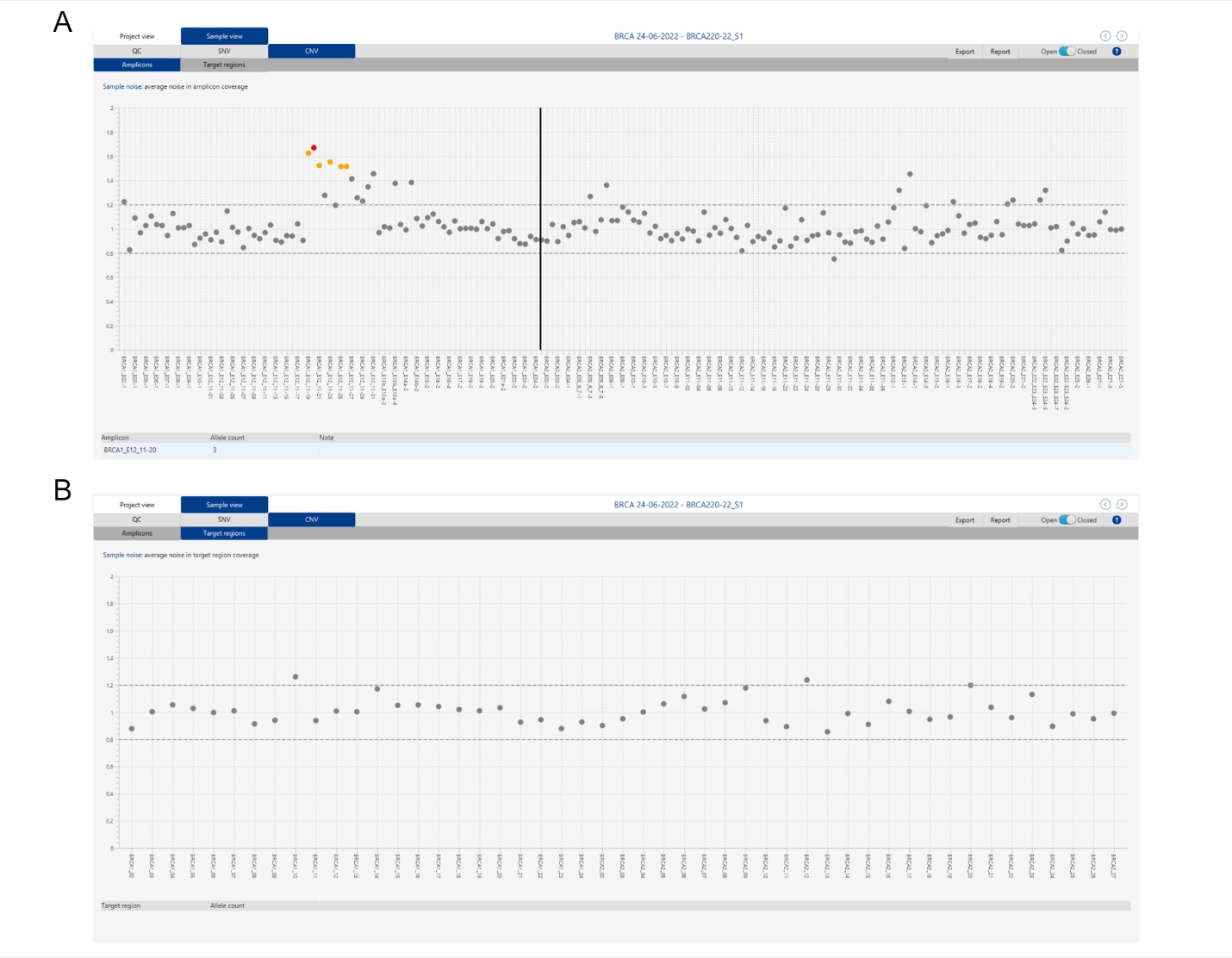


**Supplementary Figure 1: CNVs analysis performed using CE-IVD NGS BRCA Devyser kit.** A) CNV analysis for single amplicons. B) CNV analysis for target region. This analysis was performed using Amplicon Suite Software. The images show the graphic representation of the copy number values found for the different amplicones/target regions considered. Grey dots represent non-pathological oscillation (0.70 ≤ CNV ≤ 1.50); orange dots represent a deviation from the normal fluctuation of values as a borderline situation between normality and possible duplication (1.50 < CNV < 1.66); red dots indicate a possible duplication (CNV ≥ 1.66).

**
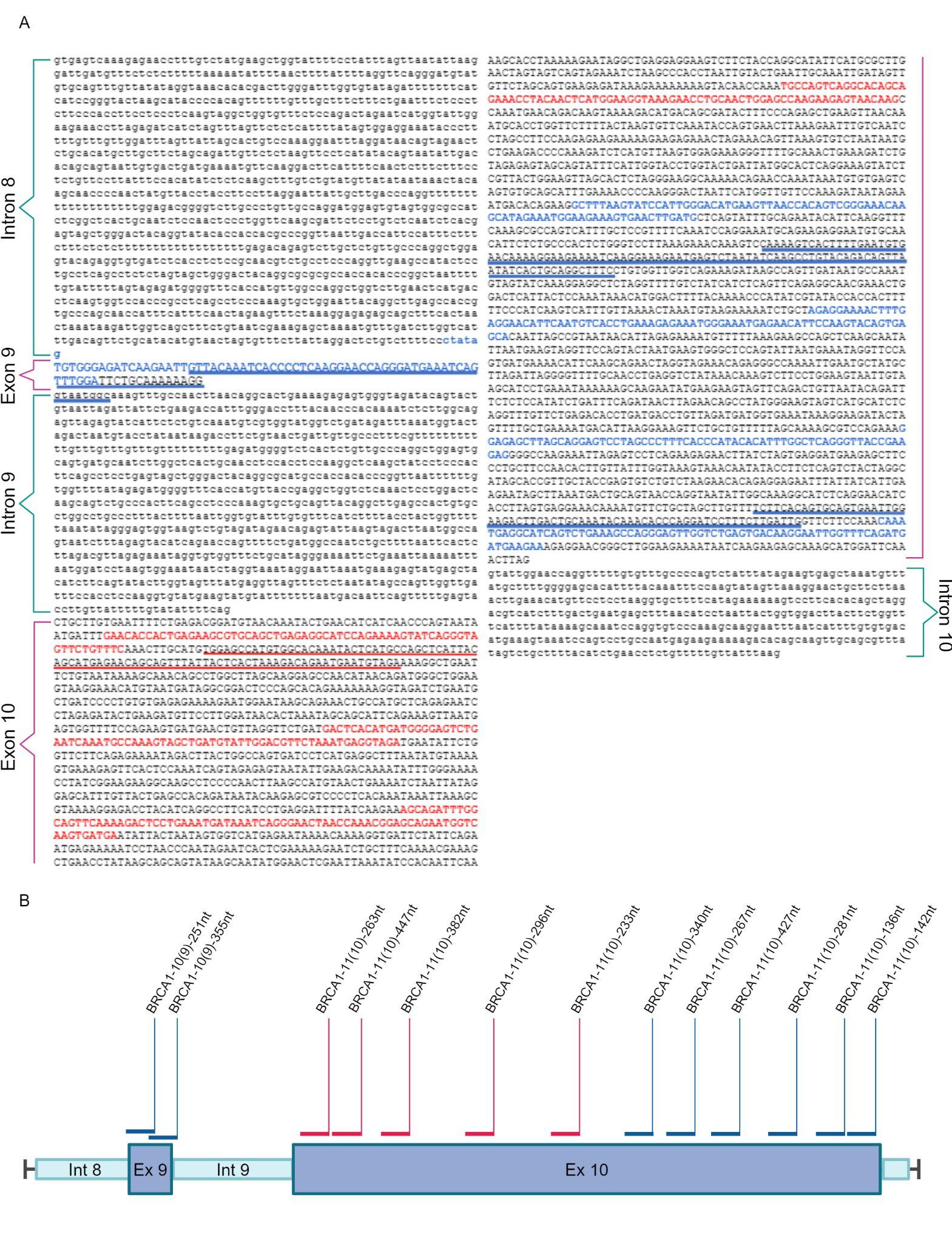
**

**Supplementary Figure 2: MLPA analysis.** (A) The panel shows the *BRCA1* partial sequence where the MLPA probes sequence has been reported. The blue colour indicates probes with a wild-type copy number, whereas the red one indicates probes with an altered copy number. The coloured sequences refer to MLPA P002 D1 probes, whereas the underlined sequences refer to MLPA P087 D1. (B) A schematic representation of *BRCA1* exons 9 and 10 and MLPA probes distribution is shown. Blue lines represent wild-type probes, whereas the red ones refer to altered probes. The name probes show the *BRCA1* exons number following the legacy numbering, the current exons numbering is in brackets.

**
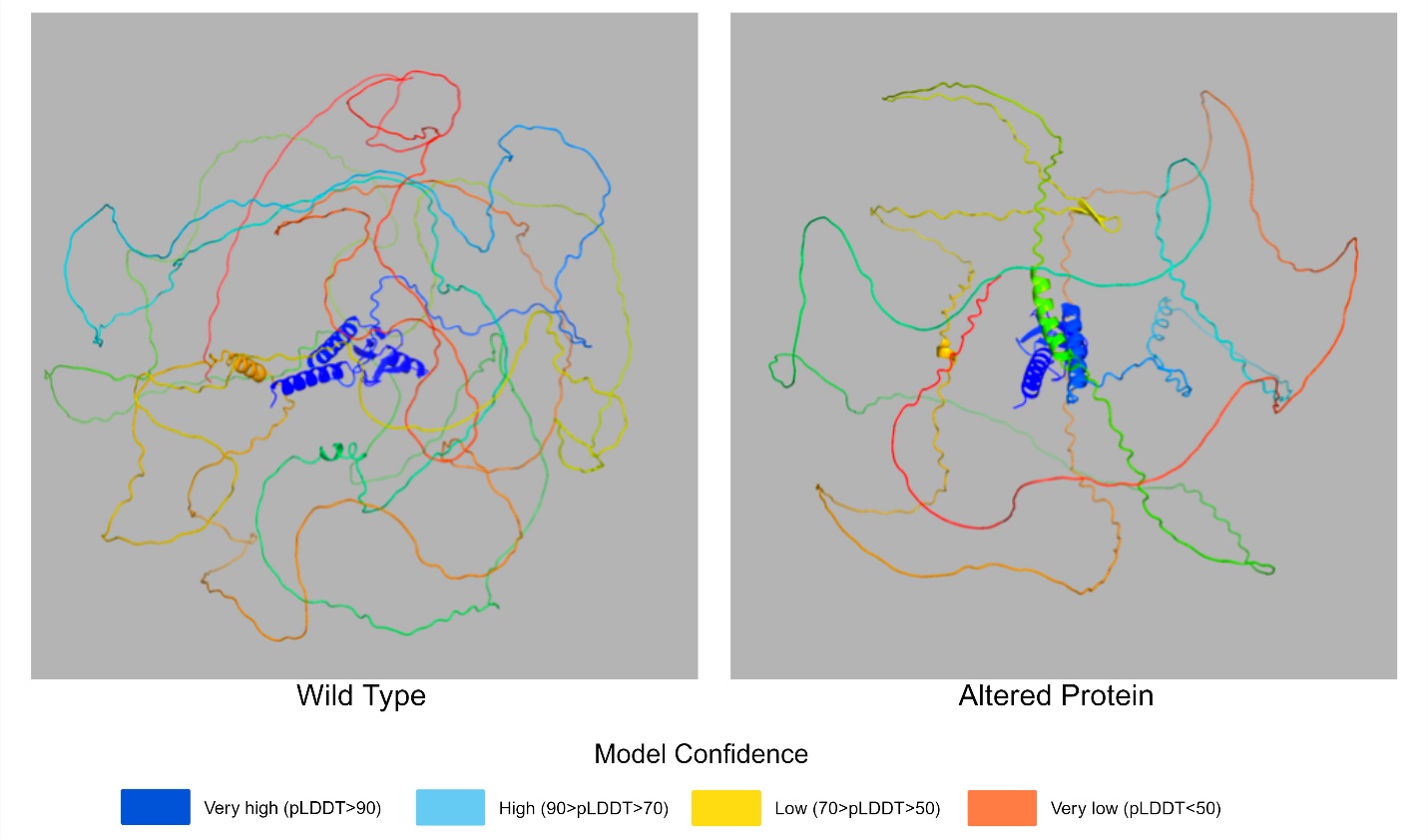
**

**Supplementary Figure 3:** The wild type amino acid sequence, 1863 amino acids long, was analyzed on the AlphaFold platform (https://alphafold.ebi.ac.uk), where it was previously resolved by DeepMind and is identified by the code AF-P38398-F1-v4. This file is available at https://www.alphafold.ebi.ac.uk/entry/A0A386IPY7. The mutated gene amino acid sequence, consisting of 771 amino acids, was also searched on AlphaFold but had not been resolved. Therefore, we processed it using AlphaFold via the Colab notebook provided by DeepMind. The mutated protein was resolved with the notation p.(Ser763Leufs*10), and the wildtype protein was retrieved from the UniProt portal.

The color-coded structure reflects model confidence values across the protein. High-confidence regions are shown in blue, while low-confidence regions transition to yellow, orange, and red. Green represents moderate-confidence areas, typically indicating intermediate values in the confidence spectrum. This gradient allows for a visual differentiation of confidence levels, where colder colors (blues) suggest higher confidence and warmer colors (reds/oranges) indicate lower reliability in the model’s predictions.


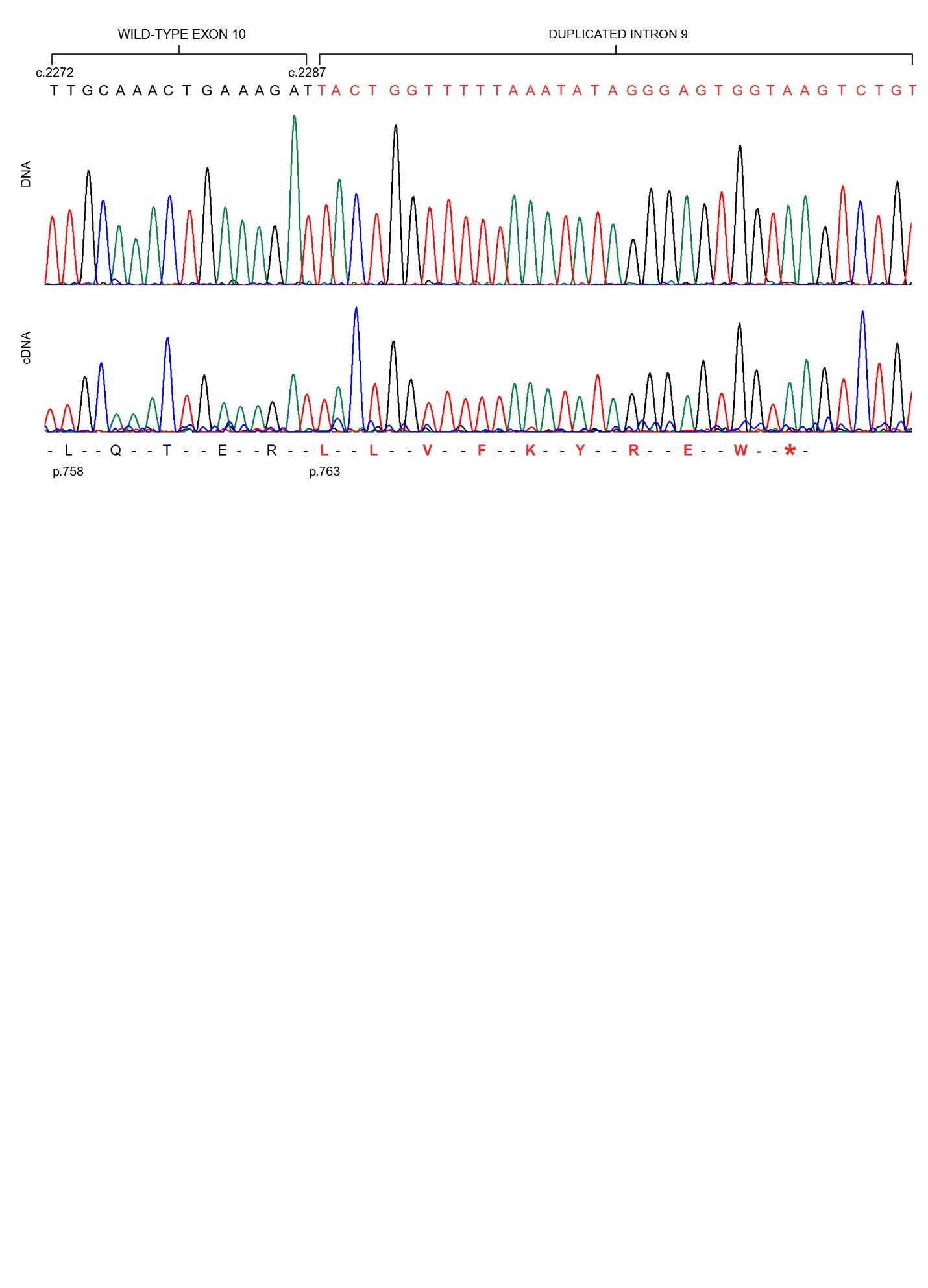


**Supplementary Figure 4:** The patient’s DNA and cDNA chromatograms show the wild-type exon 10 nucleotides and the insertion of the duplicated nucleotides. The figure highlights the breakpoint at DNA and cDNA level and the formation of a premature stop codon after 10 residues from the insertion site. *Stop codon.

**Supplementary Table 1. Experimental conditions for cDNA PCR**. T_a_: the annealing temperature of the experimental protocols.

| **GENE** | **FORWARD PRIMER 5’-3’** | **EXONS** | **REVERSE PRIMER 5’-3’** | **T_a_** | **CYCLES** |
| --- | --- | --- | --- | --- | --- |
| *BRCA1* | GAGCCAAGAAGAGTAACAAGCC | 10 | CAGACTCCCCATCATGTGAG | 62°C | 35 |
| *BRCA1* | CTTCCCTGCTTCCAACACTT | 10-11 | TCACTCTCACACCCAGATGC | 59°C | 35 |

**Supplementary Table 2. Results of the *in silico* assessment of the variant pathogenicity.**

| **Software** | **Pathogenicity Probability** |
| --- | --- |
| ClassifyCNV | 0 |
| ISV_CNV | 0.045 |
| StrVctVre | 0.533 |

|  |
| --- |
